# Supplementary material for: Nontuberculous mycobacteria remodel lung microbiota in cystic fibrosis-associated respiratory infections
Source: Microbiol Spectr. 2025 Jul 31;13(9):e00382-25. doi: 10.1128/spectrum.00382-25 (PMC12403607; doi:10.1128/spectrum.00382-25)
Supplement: Tables S1 to S3 — Species associations. [file spectrum.00382-25-s0001.docx]

Supplementary material for: Non-tuberculosis mycobacteria remodel lung microbiota in cystic fibrosis associated respiratory infections.

Michelle Hardman, Sarah Higgi, Liam Hanson, Kristin Schutz, Matthew J. Wargo, Charlotte C. Teneback, Thomas W. V. Daniels, Christopher van der Gast*, & Damian W. Rivett*

**Table S1** Multilevel pattern analysis of indicator species associated with antibiotic treatment.

| Taxonomy^a^ | Test Statistic | p value |
| --- | --- | --- |
| *Capnocytophaga gingivalis*^b^ | 0.385 | 0.050 |
| *Fusobacterium nucleatum*^b^ | 0.430 | 0.015 |
| *Lachnoanaerobaculum saburreum* | 0.486 | 0.045 |
| *Lancefieldella parvula*^b^ | 0.544 | 0.040 |
| *Prevotella intermedia* | 0.385 | 0.050 |
| *Prevotella pallens* | 0.430 | 0.020 |
| *Prevotella salivae*^b^ | 0.510 | 0.030 |
| *Streptococcus salivarius* | 0.755 | 0.005 |
| *Streptococcus thermophilus*^b^ | 0.425 | 0.040 |

^a^Given the length of the ribosomal sequences analysed, species identities should be considered putative.

^b^Significant associations with one or more other groups and therefore removed to reduce bias.

**Table S2** Multilevel pattern analysis of indicator species associated (higher frequency and abundance) with either the NTM negative or NTM positive cohorts.

| Association | Taxonomy^a^ | Test Statistic | p value |
| --- | --- | --- | --- |
| NTM negative | *Gemella morbillorum* | 0.518 | 0.020 |
|  | *Megasphaera micronuciformis* | 0.471 | 0.005 |
|  | *Prevotella histicola* | 0.421 | 0.035 |
|  | *Pseudomonas aeruginosa* | 0.779 | 0.005 |
|  | *Schaalia odontolytica* | 0.445 | 0.050 |
|  | *Selenomonas felix* | 0.385 | 0.040 |
|  | *Stomatobaculum longum* | 0.428 | 0.040 |
|  | *Streptococcus intermedius* | 0.414 | 0.045 |
|  | *Veillonella nakazawe* | 0.654 | 0.005 |
|  | *Veillonella rogosae* | 0.365 | 0.045 |
| NTM positive | *Achromobacter xylosoxidans* | 0.408 | 0.035 |

^a^Given the length of the ribosomal sequences analysed, species identities should be considered putative.

**Table S3** Multilevel pattern analysis of indicator species associated (higher frequency and abundance) with either the NTM negative or NTM positive cohorts.

| Association | Taxonomy^a^ | Test Statistic | p value |
| --- | --- | --- | --- |
| NTM negative, no modulator | *Enterococcus faecalis* | 0.447 | 0.035 |
|  | *Lactobacillus acidophilus* | 0.471 | 0.035 |
| NTM negative, modulator | *Gemella morbillorum* | 0.616 | 0.005 |
|  | *Megasphaera micronuciformis* | 0.577 | 0.010 |
|  | *Prevotella histicola* | 0.509 | 0.030 |
|  | *Stomatobaculum longum* | 0.522 | 0.015 |
|  | *Streptococcus intermedius* | 0.505 | 0.030 |
| NTM positive, no modulator | *Stenotrophomonas maltophilia* | 0.465 | 0.050 |
|  | *Achromobacter xylosoxidans* | 0.432 | 0.035 |
| NTM negative | *Pseudomonas aeruginosa* | 0.762 | 0.005 |
| No modulator | *Burkholderia cepacia* complex | 0.656 | 0.01 |
|  | *Staphylococcus succinus* | 0.614 | 0.015 |
|  | *Haemophilus influenzae* | 0.564 | 0.045 |
| Modulator | *Peptostreptoccus stomatis* | 0.516 | 0.050 |
|  | *Veillonella dispar* | 0.516 | 0.030 |
| NTM negative & NTM positive with modulator | *Veillonella nakazawae* | 0.695 | 0.005 |
|  | *Streptococcus symci* | 0.688 | 0.005 |

^a^Given the length of the ribosomal sequences analysed, species identities should be considered putative.
